# Excess Alcohol‐Induced Hospitalisations and Deaths During the First Year of the COVID‐19 Pandemic in Australia

**DOI:** 10.1111/dar.14097

**Published:** 2025-06-29

**Authors:** Wing See Yuen, Nicola Man, Michael Livingston, Agata Chrzanowska, William Gilmore, Louise Tierney, Lauren Moran, Amy Peacock

**Affiliations:** ^1^ National Drug and Alcohol Research Centre UNSW Sydney Sydney Australia; ^2^ National Drug Research Institute, enAble Institute, Faculty of Health Sciences, Curtin University Perth Australia; ^3^ Centre for Alcohol Policy Research La Trobe University Melbourne Australia; ^4^ The Burnet Institute Melbourne Australia; ^5^ Department of Clinical Neuroscience Karolinska Institute Stockholm Sweden; ^6^ Australian Institute of Health and Welfare Canberra Australia; ^7^ Australian Bureau of Statistics Canberra Australia; ^8^ School of Psychology University of Tasmania Hobart Australia

**Keywords:** alcohol, alcohol‐related harm, COVID‐19, hospitalisation, mortality

## Abstract

**Introduction:**

Since the onset of COVID‐19, alcohol‐related harm has increased in regions such as the United States and the United Kingdom. We examined whether alcohol‐related harm increased with the pandemic in Australia, and whether the impact varied across sex, age and type of alcohol diagnosis.

**Methods:**

Monthly rates of alcohol‐induced hospitalisations and deaths nationally from July 2016 until February 2020 were modelled in an autoregressive integrated moving average analysis, and the counterfactual trend was forecasted until April 2021. We estimated the overall excess in average monthly numbers of alcohol‐induced hospitalisations and deaths by sex, age and diagnosis.

**Results:**

We found excess monthly alcohol‐induced hospitalisations overall (681 [95% prediction interval 481–872]), among males (437 [343–528]), females (208 [50–355]), 15‐ to 34‐year‐olds (144 [57–226]), 35‐ to 54‐year‐olds (331 [2–636]), for cardiovascular, digestive and endocrine diseases (164 [108–223]) and for neuropsychiatric conditions (483 [236–721]). Excess monthly alcohol‐induced deaths were found overall (13 [4–21]), among males (10 [0–19]), females (4 [1–7]), 35‐ to 54‐year‐olds (8 [5–11]) and for cardiovascular, digestive and endocrine diseases (10 [2–18]) and poisonings (2 [0–4]).

**Discussion and Conclusions:**

Increased alcohol‐induced hospitalisations and deaths across Australia indicate a need to continue to monitor the long‐term impacts of the COVID‐19 pandemic and develop strategies to minimise further harm among those currently affected and in the event of future public health crises.

## 1 Introduction

Since the onset of the 2019 coronavirus (COVID‐19) pandemic, concerns have been raised about potential widespread changes in alcohol consumption and accompanying changes in alcohol‐related harm due to the physical and mental toll of a prolonged pandemic [1, 2]. Meta‐analyses of seven Australian studies showed that similar proportions reported increasing (28%) or decreasing (25%) their alcohol consumption in 2020, with a similar pattern observed in North America and the United Kingdom [3]. Concerningly, increases in consumption appear to be most common among people with the highest pre‐pandemic alcohol use [3, 4, 5]. As this group carries most of the health burden of alcohol, it is reasonable to expect a corresponding increase in health harms.

While the long‐term public health effects of changes in alcohol consumption may take some time to emerge, findings from countries such as Canada [6], Japan [7], Iran [8], the United Kingdom [5, 9], the United States [9] and those in Europe [10] suggest that alcohol‐induced hospitalisations and deaths have been rising since 2020, particularly among those with pre‐existing alcohol problems [5, 6]. However, a study in Germany [11] showed that hospital discharges decreased for both men and women. Studies of hospitalisations and deaths have thus far been restricted to specific alcohol‐related diagnoses (e.g., intoxications [8], liver disease [7]) or, where a wider range of alcohol‐related diagnoses was examined, to a broader clustering of all alcohol‐related diagnoses with limited to no disaggregation by diagnosis type [6, 9, 11]. This is also coupled with limited disaggregation by age (e.g., restricting estimates of change to older age groups [11], constructing very broad age groups [7, 8]). Consequently, it is difficult to discern whether any effects of the pandemic on alcohol‐related harm were localised to specific age groups and/or exacerbated specific alcohol‐related health problems. This information is crucial for health services and policymakers to inform the allocation of resources for treatment and prevention.

It is also important to consider whether there are contextual differences in how COVID‐19 impacted alcohol‐related harm. Differences in how Australia experienced the pandemic may mean that trends in alcohol‐related harm differ from patterns observed in other countries. Notably, Australia had some of the lowest rates of COVID‐19 infection and pandemic‐induced excess mortality in the world [12], with early closure of international borders feasible as an island nation, plus an initial ‘zero‐COVID’ approach including quarantine and lockdown measures [13]. Alcohol availability and opportunities to consume alcohol in Australia were also heavily impacted due to national and jurisdictional restrictions aimed at reducing COVID‐19 transmission (e.g., nighttime curfews, restrictions on large gatherings, people not being able to remove masks to drink alcoholic beverages in public) [14]. On the other hand, widespread disruptions to alcohol and other drug (AOD) treatment services across Australia [15, 16] may have exacerbated harm among people with pre‐existing risky alcohol use and prevented people with emerging problems from seeking help. To our knowledge, Australian research has been concentrated on changes in alcohol‐related harms at the local or state/territory level (e.g., [17, 18]), with a notable lack of information on national trends. Detailed examination of changes to alcohol‐induced hospitalisation and death rates, including disaggregation by sex, age and type of diagnosis, is needed to understand how the COVID‐19 pandemic has impacted alcohol‐related harm.

As such, the aims of this study were to:

1. Examine whether the trend in the monthly rate of alcohol‐induced hospitalisations and deaths changed with the onset of the COVID‐19 pandemic and associated restrictions in Australia until April 2021.
2. Examine whether the trend varied by sex, age and type of alcohol diagnosis.

## 2 Methods

### 2.1 Data Sources

#### 2.1.1 National Hospital Morbidity Database (NHMD)

The NHMD is a collection of electronic confidentiality summary records for completed episodes of care in all public and private hospitals in Australia, provided by the Australian Institute of Health and Welfare. Diagnoses in the NHMD are coded according to the International Classification of Diseases and Related Health Problems, Tenth Revision Australian Modification (ICD‐10‐AM). Alcohol‐induced hospitalisations defined by a principal diagnosis that was fully attributable to alcohol [19] were extracted (see Table A1 in Appendix A for the ICD codes defining alcohol‐induced hospitalisations).

Hospitalisations with a length of stay longer than 60 days were excluded as admission month was not provided for these longer hospitalisations for the purpose of confidentialisation of records.

#### 2.1.2 Australian Bureau of Statistics (ABS) Causes of Death Unit Record File

The ABS data for Causes of Death comprise deaths that occurred in Australia. These data were collated by the ABS from the Registries of Births, Deaths and Marriages in each state or territory as well as the National Coronial Information System. Our data extract comprised those deaths registered by the end of 2022. Alcohol‐induced deaths were identified by the presence of any alcohol‐induced ICD‐10 codes as defined by the ABS [20] in the underlying cause of death (see Table A2 in Appendix A for ICD codes defining alcohol‐induced deaths).

### 2.2 Study Design

This was a time‐series analysis of alcohol‐induced hospitalisations and deaths aggregated into monthly intervals. We adopted a counterfactual forecast approach, meaning that observed events during the pandemic (i.e., from March 2020) were compared to forecasted events based on the pre‐pandemic time series. This approach was chosen because multiple changes in policies on travel and gathering restrictions during the COVID‐19 pandemic made it difficult to determine the additional breakpoints for analysis, such as an interrupted time series or a segmented regression analysis [14].

The earliest date of 1 July 2016 was chosen as monthly data on all‐cause hospitalisation rates was used in the analyses to account for the impact of the pandemic on hospital services, and these data were only available from this date [21]. Data were available for deaths recorded on or before 31 December 2022 and for hospitalisations ending on or before 30 June 2021. Therefore, April 2021 was the latest month that data for all hospital admissions within the scope of the study (i.e., ≤ 60 days in length) were available. There could sometimes also be a lag in registration of deaths. For these reasons, we truncated the data series on 30 April 2021 for alcohol‐induced hospitalisations and deaths to minimise bias.

We only included records from individuals aged ≥ 15 years at the time of hospital admission or death because the onset of drinking behaviour typically starts from around 15 years onwards [22].

Findings are reported according to the Strengthening the Reporting of Observational Studies in Epidemiology (STROBE) and REporting of studies Conducted using Observational Routinely‐collected health Data (RECORD) reporting guidelines (see Table B1) [23, 24].

### 2.3 Measures

Our primary outcome measures were the average monthly difference between the observed and the expected number of alcohol‐induced hospitalisations and deaths. Note that we used the term ‘alcohol‐induced’ to describe hospitalisations and deaths that were wholly attributable to alcohol, whereas the term ‘alcohol‐related’ as used in the Introduction and Discussion includes harms that were partly or wholly attributable to alcohol. The outcome measures were disaggregated by sex (male and female), age group at date of hospital admission or date of death (15–34, 35–54 and ≥ 55 years) and type of alcohol diagnosis. Alcohol diagnosis type was identified based on the principal diagnosis for alcohol‐induced hospitalisations and the underlying cause of death for alcohol‐induced deaths. We focused on three broad categories of alcohol diagnoses: (i) alcohol poisoning, (ii) neuropsychiatric conditions, and (iii) cardiovascular, digestive and endocrine (CDE) diseases (see Table A3 in Appendix A for codes). Rates were calculated using the quarterly estimated national resident population data at the end of March, June, September and December of each year as the population denominator for the January–March, April–June, July–September and October–December quarters, respectively, as monthly data were not available [25].

### 2.4 Data Analysis

#### 2.4.1 Primary Analysis

This was an exploratory study; hence, the analyses were not pre‐registered. Autoregressive integrated moving average (ARIMA) models were fitted to the time series data from July 2016 until February 2020 for overall rates of alcohol‐induced hospitalisations and deaths and rates by sex, age and diagnosis type. Of the 352,269 alcohol‐induced hospital admissions identified, 17 records were missing data on sex, and 2308 records were missing data on age; these cases were excluded for analyses by age and sex, respectively. Monthly data on all‐cause hospitalisation rates was fitted as a covariate in the ARIMA model for hospitalisation rates because reports indicate that hospital services were affected by the COVID‐19 pandemic [21]. February 2020 was chosen as the last time point for the pre‐pandemic period because the World Health Organization's declaration of the COVID‐19 pandemic was on 11 March 2020 [26], and the start of Australia's nationwide restrictions occurred towards the end of March 2020 [14]. The time series analyses were performed using the *fable* package in *R* [27, 28]. The default automated algorithm in the *fable* package was used to determine the ARIMA model of best fit [29].

Forecast of the counterfactual trend over time was then performed using the ARIMA model for each time series until 31 April 2021. To account for non‐independence of data and potential non‐normality of the residuals, the residuals were bootstrapped (using the bootstrap option in the forecast function) to produce 40,000 samples from which the forecast estimates and corresponding prediction intervals (PIs) were computed. For comparison of the counterfactual trend with the observed rate in the plots, 80% and 95% PIs were produced from the bootstrap samples. The difference between the observed rates from the counterfactual estimates and the observed rates was back‐transformed and averaged for each time series to obtain a pooled estimate of excess or deficit hospitalisations and deaths per month and corresponding 95% PI. The pooled estimate of excess or deficit hospitalisations and deaths per month was deemed to be significantly different from 0 if the 95% PI did not include 0.

Model assumptions were assessed with unit root tests, histogram autocorrelation and partial autocorrelation plots.

#### 2.4.2 Sensitivity Analyses

A sensitivity analysis for alcohol‐induced hospitalisation rates without adjustment for all‐cause hospitalisation rates was also conducted to assess whether the adjustment could affect the results.

### 2.5 Ethics

Ethics approval for the study and use of the data was provided by the UNSW Human Research Ethics Committees (HREC; HC220754), the Royal Melbourne Hospital HREC (HREC/101690/MH‐2023) and the SA Department for Health and Wellbeing HREC (2020/HRE0043).

## 3 Results

### 3.1 Overall and by Sex

**FIGURE 1:**
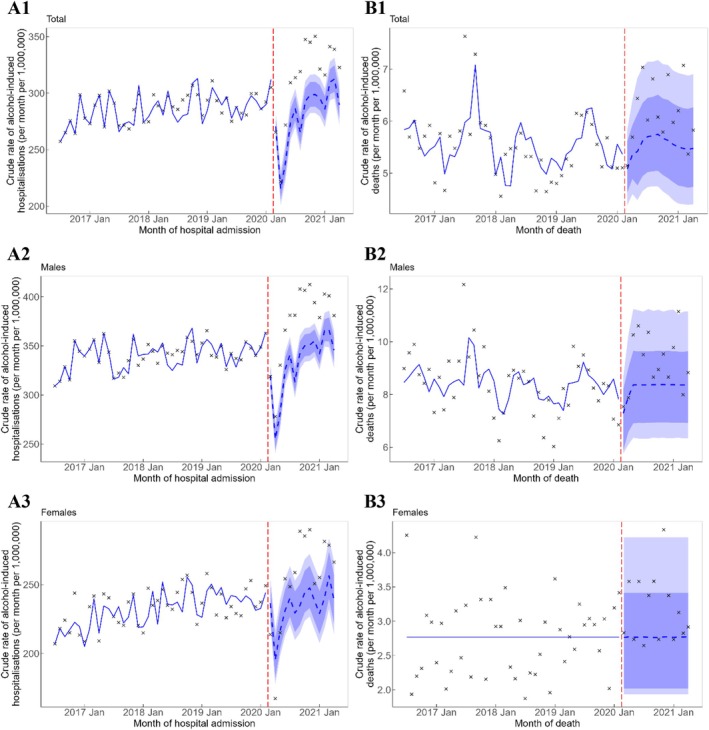
Crude rate (per month per 1,000,000 persons) of alcohol‐induced hospitalisations (A) and deaths (B)—overall (A1 and B1) and by sex (A2, A3, B2 and B3). Vertical red dashed line represents the onset of the COVID‐19 pandemic. Crosses represent observed rates. Blue solid line represents the estimated trend, and blue dashed line represents the counterfactual forecast of the trend after the onset of the COVID‐19 pandemic. Light blue and dark blue bands represent the 80% and 95% prediction intervals, respectively, of the counterfactual forecast of the rates. (A1) Overall, hospitalisations; (A2) Males, hospitalisations; (A3) Females, hospitalisations; (B1) Overall, deaths; (B2) Males, deaths; and (B3) Females, deaths.

Based on the pooled estimates from the counterfactual forecast, there was a significant excess in overall alcohol‐induced hospitalisations of 681 admissions per month (95% PI = 481, 872) above what was predicted during the COVID‐19 pandemic in this study period (Table 1). There was also a significant excess of hospitalisations per month for both sexes (Table 1), with 437 (95% PI = 343, 528) and 208 (95% PI = 50, 355) admissions per month above what was predicted among males and females, respectively (Table 1). The observed rates of alcohol‐induced hospitalisation were consistently above the 95% PI from the counterfactual analysis for the overall population and among males from June 2020 until the end of the study, while the majority of observed alcohol‐induced hospitalisation rates among females were above the 80% PI (Figure 1).

**TABLE 1:** Average difference in number of alcohol‐induced hospitalisations and deaths per month between the observed and the counterfactual forecast in the COVID‐19 pandemic period (March 2020 to April 2021).

	Alcohol‐induced hospitalisations	Alcohol‐induced deaths
	Estimate (95% PI)	Estimate (95% PI)
Overall	681 (481, 872)	13 (4, 21)
Sex		
Males	437 (343, 528)	10 (0, 19)
Females	208 (50, 355)	4 (1, 7)
Age		
15–34 years	144 (57, 226)	1 (0, 2)
35–54 years	331 (2, 636)	8 (5, 11)
55+ years	129 (−47, 305)	2 (−6, 9)
Diagnosis type		
CDE diseases	165 (108, 223)	10 (2, 18)
Neuropsychiatric	483 (236, 721)	1 (−4, 6)
Poisoning	−3 (−10, 4)	2 (0, 4)

*Note*: Bolded rows indicate estimates with 95% PIs that do not include 0.

Abbreviations: CDE, cardiovascular, digestive and endocrine; PI, prediction interval.

The observed rate of alcohol‐induced deaths in the overall population and by sex was generally higher than the counterfactual trend (the blue line from March 2020 onwards in Figure 1), and even though the majority of the observed data were within the 80% PI, the pooled estimates indicate that there were significant excess alcohol‐induced deaths, overall (13 deaths per month [95% PI = 4, 21]) and by males and females (10 [95% PI = 0, 19] and 4 [95% PI = 1, 7] deaths per month, respectively).

### 3.2 Age Group

**FIGURE 2:**
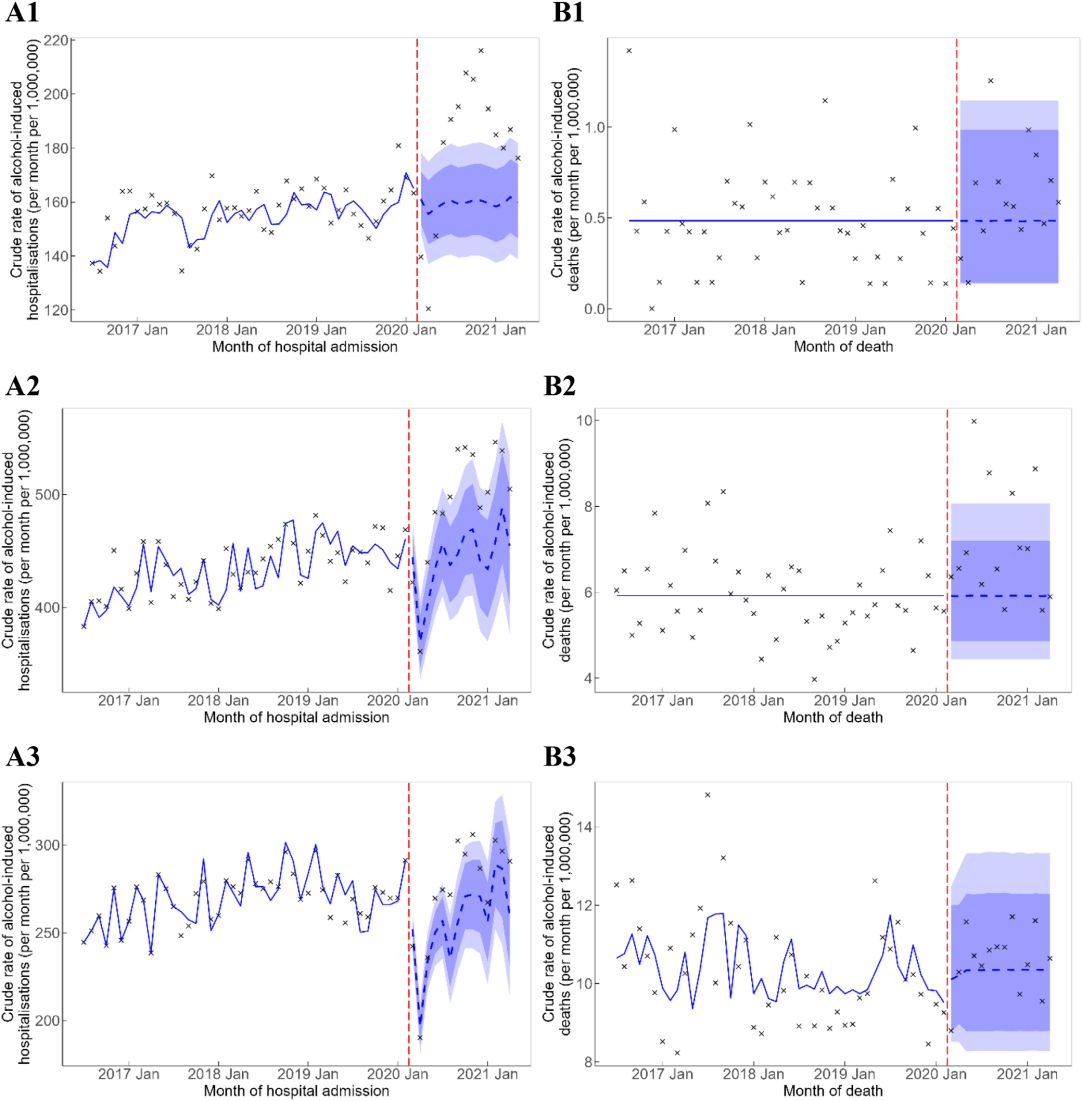
Crude rate of alcohol‐induced hospitalisations (A1–A3) and deaths (B1–B3) by age group. Vertical red dashed line represents the onset of the COVID‐19 pandemic. Crosses represent observed rates. Blue solid line represents the estimated trend, and blue dashed line represents the counterfactual forecast of the trend after the onset of the COVID‐19 pandemic. Light blue and dark blue bands represent the 80% and 95% prediction intervals, respectively, of the counterfactual forecast of the rates. (A1) 15–34, hospitalisations; (A2) 35–54, hospitalisations; (A3) 55 and over, hospitalisations; (B1) 15–34, deaths; (B2) 35–54, deaths; and (B3) 55 and over, deaths.

There was a significant excess in alcohol‐induced hospitalisations among people aged 15–34 years (144 admissions per month [95% PI = 57, 226]) as well as excess alcohol‐induced hospitalisations and deaths among people aged 35–54 years (331 admissions [95% PI = 2, 636] and 8 deaths [95% PI = 5, 11] per month, respectively). Figure 2 shows that the observed hospitalisation rate among people aged 15–34 years was above the 95% PI between July and December 2020, and the majority of the observed rates were above the 80% PI from around August 2020 onwards for people aged 35–54 years. The deviation of observed rates from the counterfactual trend for alcohol‐induced deaths among people aged 35–54 years of age was, however, less clear even though the majority of observed alcohol‐induced death rates were above the counterfactual trend.

### 3.3 Diagnosis Type

**FIGURE 3:**
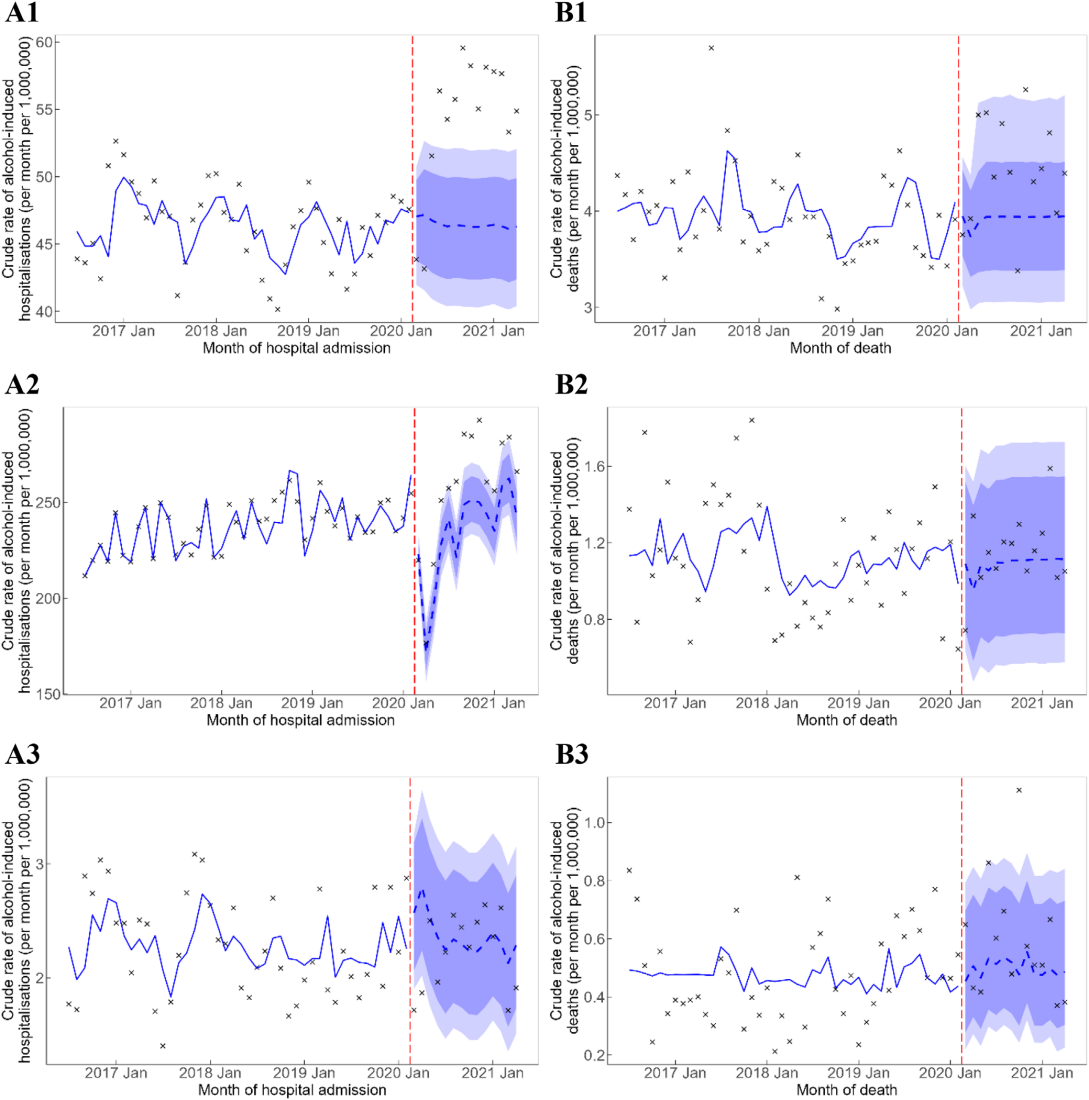
Crude rate of alcohol‐induced hospitalisations (A1–A3) and deaths (B1–B3) by major alcohol involvement. Vertical red dashed line represents the onset of the COVID‐19 pandemic. Crosses represent observed rates. Blue solid line represents the estimated trend, and blue dashed line represents the counterfactual forecast of the trend after the onset of the COVID‐19 pandemic. Light blue and dark blue bands represent the 80% and 95% prediction intervals, respectively, of the counterfactual forecast of the rates. CDE, cardiovascular, digestive and endocrine. (A1) CDE diseases, hospitalisations; (A2) Neuropsychiatric, hospitalisations; (A3) Poisoning, hospitalisations; (B1) CDE diseases, deaths; (B2) Neuropsychiatric, deaths; and (B3) Poisoning, deaths.

There were significant excess alcohol‐induced hospital admissions per month with diagnoses relating to CDE diseases (165 [95% PI = 108, 223]) and neuropsychiatric conditions (483 [95% PI = 236, 721]). The observed rate of alcohol‐induced hospitalisation with a diagnosis for CDE diseases and neuropsychiatric conditions from June 2020 onwards was consistently above the 80% PI (Figure 3). There was an estimated excess of 10 (95% PI = 2, 18) and 2 (95% PI = 0, 4) alcohol‐induced deaths from CDE diseases and alcohol poisoning, respectively, during the COVID‐19 pandemic. The observed rate of alcohol‐induced deaths from CDE diseases was mostly higher than the counterfactual trend except for the October 2020 data point. However, observed rates of alcohol‐induced poisoning deaths were low, and deviation from the counterfactual trend could not be clearly discerned.

### 3.4 Sensitivity Analysis for Alcohol‐Induced Hospitalisations

**FIGURE C1:**
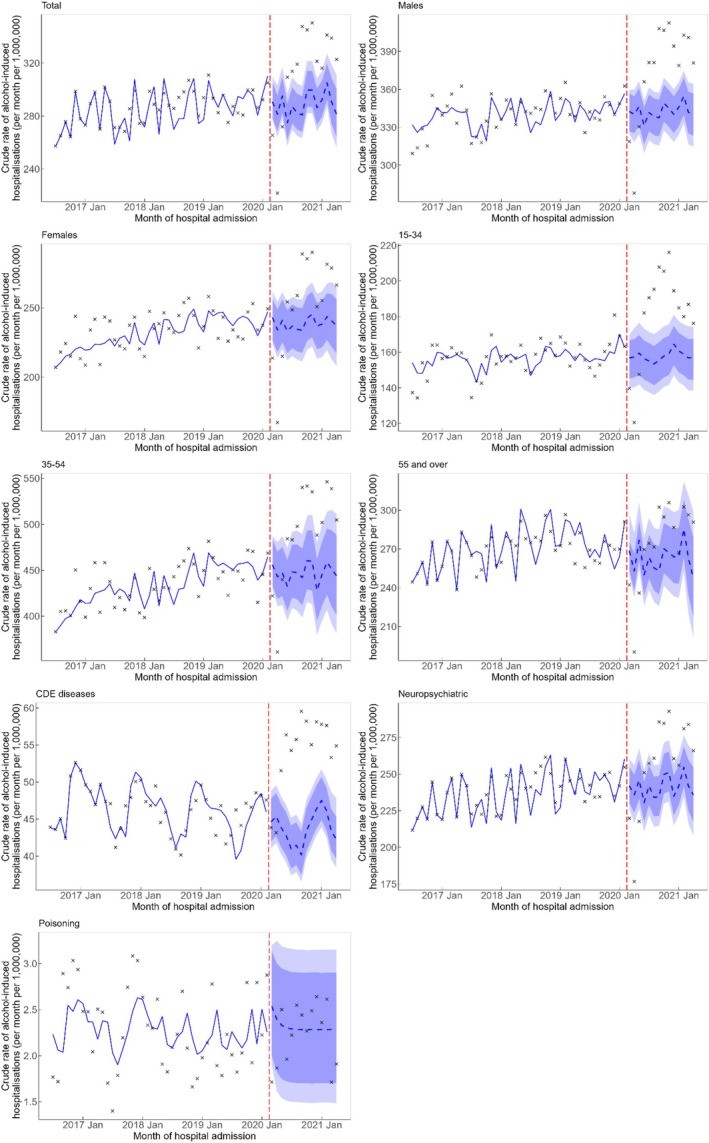
Crude rate (per month per 1,000,000 persons) of alcohol‐induced hospitalisations (without adjustment for all‐cause hospitalisation rates).

In the sensitivity analysis (Table C1 and Figure C1 in Appendix C), where we did not adjust for all‐cause hospitalisations, the direction and significance of the estimates of excess alcohol‐induced hospitalisations were the same as in the main analysis. The estimates of excess hospitalisations from the main results were outside of the 95% PI from the sensitivity analysis and/or vice versa for the overall estimate and for males and CDE diseases. The main estimates were higher for the overall number of hospitalisations and for males, while they were lower for hospitalisations with CDE diseases.

## 4 Discussion

We found an overall excess of both alcohol‐induced hospitalisations and deaths in Australia after the onset of the COVID‐19 pandemic, which was observed among both males and females. Results differed by age, with an excess in alcohol‐induced hospitalisations among people aged 15–54 years and an excess in alcohol‐induced deaths among people aged 35–54 years. Among people aged 15–34 years, alcohol‐induced hospitalisations were particularly elevated during July to December 2020. Findings also differed by diagnosis type, with an excess in alcohol‐induced hospitalisations and deaths coded as CDE diseases, and in alcohol‐induced hospitalisations coded as neuropsychiatric conditions. While there was also a statistically significant excess in alcohol‐induced poisoning deaths, rates remained relatively low. Overall, excess alcohol‐induced hospitalisations and deaths were more evident from mid‐2020 onwards.

Comparable studies from Canada [6], Germany [11], Japan [7], Iran [8], the United Kingdom [9] and the United States [9] have reported increases in alcohol‐induced hospitalisations and deaths. Previous studies have had limited disaggregation by age and range of diagnoses, and there are no other studies to our awareness that examine changes in alcohol‐related harms nationally with the onset of COVID‐19. Our Australian findings were consistent with overall trends in alcohol‐induced hospitalisations and deaths in other countries [6, 7, 8, 9, 11], indicating the widespread impact of COVID‐19 on alcohol‐related harm despite differences in research context and method (e.g., COVID‐19 transmission rates, public health measures, alcohol use culture, time period, diagnosis codes). In particular, we found that alcohol‐induced hospitalisations increased irrespective of systemic service use changes resulting from the pandemic.

Though we identified a decrease (or lack of increase) in alcohol‐induced hospitalisations in the first few months of the pandemic, which may have been a real reduction in harm due to government restrictions on licensed venues and social gatherings [14], this decline was only temporary as subsequent rates were substantially higher compared to pre‐pandemic levels. The sharp drops in hospitalisations in March and April 2020 may also reflect reduced service use due to COVID‐19 restrictions and avoidance of care due to concerns about the pandemic [30].

Both the 15–34 and 35–54 age groups showed an excess of alcohol‐induced hospitalisations, peaking around August 2020. This aligns with the easing of restrictions for licensed venues and social gatherings at the beginning of June 2020 in most jurisdictions across Australia [14]. Given the duration of sustained heavy alcohol use required for chronic alcohol problems to manifest, the full scope of the health consequences stemming from pandemic‐induced shifts in alcohol consumption among young people is likely yet to emerge. In light of these excess alcohol‐induced hospitalisations in people aged 15–54 years, in addition to excess alcohol‐induced mortality among people aged 35–54 years, there is a clear need to prioritise strategies aimed at reducing alcohol‐related harm in these age groups to prevent further escalation of health problems and mortality.

The excess of both alcohol‐induced CDE and neuropsychiatric hospitalisations since the onset of the COVID‐19 pandemic was consistent with other countries such as the United States [31, 32] and Japan [7]. Pandemic‐induced increases in alcohol use among people with the highest pre‐pandemic alcohol use [3, 4, 33, 34] combined with reductions in access to AOD treatment services [15, 16, 35] mean that people who were already at high risk of alcohol‐related harm had fewer avenues of seeking help and were thus at greater risk of experiencing harm. Additionally, many adults have reported reduced physical activity since the beginning of the pandemic [36, 37, 38], compounding the risk of CDE diseases and other health problems [39].

Overall, the excess of alcohol‐induced hospitalisations and deaths in Australia from April 2020 to April 2021 validates the concerns raised early in the pandemic that there would be an increase in alcohol‐related harm [1, 2]. Deficits in AOD treatment services [15, 16, 35] are likely to have resulted in more people being hospitalised for alcohol‐induced reasons, whereas prior to the pandemic, these issues could have been addressed and minimised. As alcohol‐induced hospitalisations are known to increase the risk of subsequent hospitalisations and death due to both alcohol‐related and unrelated reasons [40, 41], the true extent of the health impact of these increases in alcohol‐related harm is yet to be determined. These alcohol‐induced health issues are also likely to be exacerbated due to co‐occurring mental health problems that have increased in tandem [36, 38, 42]. Indeed, while evidence from three Australian states suggests there was no overall increase in suicide deaths during the pandemic [43], changes in alcohol‐related harm by intent (i.e., intentional versus unintentional harm) during the pandemic are an important area for future study. While some alcohol‐related harms may have been mitigated through social distancing measures and venue restrictions, it remains evident that both alcohol‐induced hospitalisations and deaths have increased in Australia since the onset of COVID‐19. Improved regulatory measures and well‐defined action plans are imperative to minimise alcohol‐related harms during any potential future public health emergencies.

### 4.1 Limitations

This study does not use a causal inference framework, and as such, we cannot comment on the causality of COVID‐19 on rates of alcohol‐induced hospitalisations and deaths. The results from the counterfactual forecast are also dependent on the chosen ARIMA model, although we used an automated algorithm in R's fable package for model selection to minimise user bias [27, 28]. The results of the hospitalisation analyses are not a direct measure of alcohol‐related harm as rates of hospitalisations are reliant on the availability of hospital services and individuals' health‐seeking behaviours, both of which are likely to have been affected by the COVID‐19 pandemic. However, there was an excess of alcohol‐induced hospitalisations even after adjusting for all‐cause hospitalisations in our analyses. The excess of alcohol‐induced deaths is also a concrete indicator of increase in alcohol‐related harm. These increases reflect the increase in the overall amount of alcohol available for consumption reported by the Australian Institute of Health and Welfare [44] and analyses of some survey data (e.g., [45]). Although we found an excess of alcohol‐induced poisoning deaths, both alcohol‐induced poisoning hospitalisations and deaths were relatively scarce, which meant that it was difficult to detect statistically significant changes. Diagnosis‐specific analyses are also dependent on the accuracy of the attending clinician; for example, F10 may be coded for ‘complications of alcoholism’, which can refer to liver disease, ketoacidosis or another condition. Though we adjusted for all‐cause hospitalisations, these rates were not disaggregated by demographic characteristics. Estimates by sex, age and diagnosis type also do not adjust for each other, meaning that some effects may largely be explained by patterns in another characteristic of the patient (e.g., we are unable to determine whether the excess in alcohol‐induced CDE hospitalisations was driven by a particular age group).

A key limitation of this paper is its focus on national data, despite substantial differences in the experience of restrictions and COVID‐19 cases between states and territories during 2020 and 2021 [14]. It is worth noting that state/territory‐level mortality data show similar increases in alcohol‐induced deaths for most jurisdictions, with the largest increase between 2019 and 2022 occurring in Queensland, which experienced some of the lightest restrictions [46]. More work is needed to understand the jurisdictional differences in how the pandemic influenced alcohol‐related harms, but sparse data for jurisdictions with small population sizes meant we could not undertake this disaggregation. Australia experienced a net loss of 89,000 people during the 2020–2021 financial year, affecting every state and territory [47]. Trends in our estimated numbers may differ from trends in rates due to net negative migration during the pandemic.

Finally, it is important to note that our mortality data for the Years 2020 and 2021 are provisional and alcohol‐poisoning deaths may undergo revisions as additional coroner‐referred cases are closed. However, on average, 70% of alcohol‐induced deaths are certified by a doctor [20], meaning that alcohol‐induced death data are less likely to be impacted by ABS revisions and can be treated as fairly complete. Moreover, both hospitalisations and deaths data were limited to April 2021 due to lags in collating and processing these national collections. This means that the rates reported in the current study are an underestimate of alcohol‐induced hospitalisations and deaths that have occurred since the onset of COVID‐19.

## 5 Conclusions

The onset of the COVID‐19 pandemic was followed by an excess of alcohol‐induced hospitalisations and alcohol‐induced deaths across Australia. These increases in alcohol‐related harm indicate a need to continue to monitor the long‐term impacts of the COVID‐19 pandemic and develop strategies to minimise further harm, particularly among those heavily impacted (e.g., people aged 15–54 years, people with high pre‐pandemic alcohol use). Our findings emphasise the need for improved measures to mitigate alcohol‐related harm, providing a call to action to address the increased health burden since the pandemic and serving as a caution for future public health crises.

## Author Contributions

A.P., N.M. and W.S.Y. conceived the study. All authors had input on the study design. N.M. and A.C. prepared the data for analysis. N.M. conducted analyses with support from A.C. W.S.Y., N.M., M.L., A.C. and A.P. drafted the manuscript. All authors had input on revising the manuscript critically for important intellectual content. All authors approved the final version to be published. Each author certifies that their contribution to this work meets the standards of the International Committee of Medical Journal Editors.

## Conflicts of Interest

Dr Amy Peacock received untied educational grants from Seqirus and Mundipharma for study of opioid medicines in Australia. These grants have ceased, and funders had no role in study design, conduct or reporting. No pharmaceutical grants were received for the current study. Dr Michael Livingston has been a board member of the Alcohol and Drug Foundation, an Australian NGO focused on reducing harm from alcohol and other drugs, since 2023. The Alcohol and Drug Foundation, the Australian Government Department of Health and Aged Care, the Australian National Health and Medical Research Council, and the Australian Research Council had no role in study design, conduct or reporting. The other authors declare no conflicts of interest.

## Data Availability

Data may be made available on request subject to data custodian/privacy/ethical restrictions.

## Appendix A Identification of Records—Scope of Data and ICD Codes

**TABLE A1:** ICD‐10‐AM codes in principal diagnosis to identify alcohol‐induced hospitalisations.

ICD‐10‐AM code	Description
E24.4	Alcohol‐induced pseudo‐Cushing's syndrome
F10	Mental and behavioural disorders due to alcohol use
G31.2	Degeneration of nervous system due to alcohol
G62.1	Alcoholic polyneuropathy
G72.1	Alcoholic myopathy
I42.6	Alcoholic cardiomyopathy
K29.2	Alcoholic gastritis
K70	Alcoholic liver disease
K85.2	Alcohol‐induced acute pancreatitis
K86.0	Alcohol‐induced chronic pancreatitis
T51.0	Toxic effect of ethanol
T51.1	Toxic effect of methanol
T51.9	Toxic effect of alcohol, unspecified
Z71.4	Counselling and surveillance for alcohol use disorder

*Note*: Hospitalisations for which the care type was reported as ‘Newborn without qualified days’, and records for ‘Posthumous organ procurement’ and ‘Hospital boarders’ were not provided by the data custodian (Australian Institute of Health and Welfare). Cross‐border hospitalisations were also not provided in our National Hospital Morbidity Database data set, that is, the data custodian did not provide the records of patients whose state of usual residence was not the state of hospitalisation in the customised data extract.

Abbreviation: ICD‐10‐AM, International Classification of Diseases and Related Health Problems, Tenth Revision Australian Modification.

**TABLE A2:** ICD‐10 codes of causes of death attributable to alcohol‐induced mortality.

ICD‐10 codes	Description
E24.4	Alcohol‐induced pseudo‐Cushing's syndrome
F10	Mental and behavioural disorders due to alcohol use
G31.2	Degeneration of nervous system due to alcohol
G62.1	Alcoholic polyneuropathy
G72.1	Alcoholic myopathy
I42.6	Alcoholic cardiomyopathy
K29.2	Alcoholic gastritis
K70	Alcoholic liver disease
K85.2	Alcohol‐induced acute pancreatitis
K86.0	Alcohol‐induced chronic pancreatitis
X45	Accidental poisoning by and exposure to alcohol
X65	Intentional self‐poisoning by and exposure to alcohol
Y15	Poisoning by and exposure to alcohol, undetermined intent

*Note*: Alcohol‐induced causes exclude accidents, homicides, and other causes indirectly related to alcohol use. Newborn deaths associated with maternal alcohol use are also excluded.

Abbreviation: ICD‐10, International Classification of Diseases and Related Health Problems, Tenth Revision.

**TABLE A3:** Alcohol diagnosis types and corresponding ICD‐10‐AM and ICD‐10 codes used to classify the selected alcohol‐induced hospitalisations and deaths.

Alcohol diagnosis type	Alcohol‐induced hospitalisations with the selected ICD‐10‐AM codes in the principal diagnosis	Alcohol‐induced deaths with selected ICD‐10 codes in any of the associated causes of death
Alcohol poisoning	T51.0	X45
	T51.1	X65
	T51.9	Y15
Neuropsychiatric conditions	F10	
	G31.2	F10
	G62.1	G31.2
	G72.1	G62.1
	Z71.4	G72.1
Cardiovascular, digestive and endocrine diseases	E24.4	E24.4
	I42.6	I42.6
	K29.2	K29.2
	K70	K70
	K85.2	K85.2
	K86.0	K86.0

Abbreviations: ICD‐10, International Classification of Diseases and Related Health Problems, Tenth Revision; ICD‐10‐AM, International Classification of Diseases and Related Health Problems, Tenth Revision Australian Modification.

## Appendix B STROBE Checklist With the RECORD Checklist Extension

**TABLE B1:** STROBE Statement—Checklist of items in reports of cross‐sectional studies.

	Item no.	Recommendation	Section	RECORD items	Section
Title and abstract	1	(a) Indicate the study's design with a commonly used term in the title or the abstract	Abstract	RECORD 1.1: The type of data used should be specified in the title or abstract. When possible, the name of the databases used should be included.	Abstract
				RECORD 1.2: If applicable, the geographic region and timeframe within which the study took place should be reported in the title or abstract.	Abstract
		(b) Provide in the abstract an informative and balanced summary of what was done and what was found	Abstract	RECORD 1.3: If linkage between databases was conducted for the study, this should be clearly stated in the title or abstract.	N.A. (data linkage not done)
Introduction					
Background/rationale	2	Explain the scientific background and rationale for the investigation being reported	Section 1		
Objectives	3	State‐specific objectives, including any prespecified hypotheses	Section 1, last paragraph		
Methods					
Study design	4	Present key elements of study design early in the paper	Section 2.3		
Setting	5	Describe the setting, locations and relevant dates, including periods of recruitment, exposure, follow‐up and data collection	Sections 2.1 and 2.3		
Participants	6	(a) Give the eligibility criteria, and the sources and methods of selection of participants	Section 2.1	RECORD 6.1: The methods of study population selection (such as codes or algorithms used to identify subjects) should be listed in detail. If this is not possible, an explanation should be provided. RECORD 6.2: Any validation studies of the codes or algorithms used to select the population should be referenced. If validation was conducted for this study and not published elsewhere, detailed methods and results should be provided. RECORD 6.3: If the study involved linkage of databases, consider use of a flow diagram or other graphical display to demonstrate the data linkage process, including the number of individuals with linked data at each stage.	Section 2.1
Variables	7	Clearly define all outcomes, exposures, predictors, potential confounders and effect modifiers. Give diagnostic criteria, if applicable.	Section 2.4	RECORD 7.1: A complete list of codes and algorithms used to classify exposures, outcomes, confounders and effect modifiers should be provided. If these cannot be reported, an explanation should be provided.	Appendix A
Data sources/measurement	8	For each variable of interest, give sources of data and details of methods of assessment (measurement). Describe comparability of assessment methods if there is more than one group.	Section 2.1		
Bias	9	Describe any efforts to address potential sources of bias	Section 2.3		
Study size	10	Explain how the study size was arrived at	N.A. (given secondary data sources)		
Quantitative variables	11	Explain how quantitative variables were handled in the analyses. If applicable, describe which groupings were chosen and why.	Section 2.4		
Statistical methods	12	(a) Describe all statistical methods, including those used to control for confounding	Section 2.5		
		(b) Describe any methods used to examine subgroups and interactions	N.A.		
		(c) Explain how missing data were addressed	Section 2.5		
		(d) If applicable, describe analytical methods taking account of sampling strategy	Time series analysis as described in Section 2.5		
		(e) Describe any sensitivity analyses	Section 2.5		
Data access and cleaning methods				RECORD 12.1: Authors should describe the extent to which the investigators had access to the database population used to create the study population. RECORD 12.2: Authors should provide information on the data cleaning methods used in the study.	Section 2.1
Linkage				RECORD 12.3: State whether the study included person‐level, institutional‐level or other data linkage across two or more databases. The methods of linkage and methods of linkage quality evaluation should be provided.	N.A. (data linkage not done)
Results					
Participants	13	(a) Report numbers of individuals at each stage of study—for example, numbers potentially eligible, examined for eligibility, confirmed eligible, included in the study, completing follow‐up and analysed	N.A. (Individuals are not identified in our multi‐purpose data extract.)	RECORD 13.1: Describe in detail the selection of the persons included in the study (i.e., study population selection), including filtering based on data quality, data availability and linkage. The selection of included persons can be described in the text and/or by means of the study flow diagram.	N.A.
		(b) Give reasons for non‐participation at each stage			
		(c) Consider use of a flow diagram			
Descriptive data	14	(a) Give characteristics of study participants (e.g., demographic, clinical and social) and information on exposures and potential confounders	N.A.		
		(b) Indicate number of participants with missing data for each variable of interest	Section 2.4		
Outcome data	15	Report numbers of outcome events or summary measures	N.A. (irrelevant as time series analysis was performed)		
Main results	16	(a) Give unadjusted estimates and, if applicable, confounder‐adjusted estimates and their precision (e.g., 95% confidence interval). Make clear which confounders were adjusted for and why they were included.	Section 3		
		(b) Report category boundaries when continuous variables were categorised	N.A.		
		(c) If relevant, consider translating estimates of relative risk into absolute risk for a meaningful time period	Excess numbers reported as outcome measure in Section 3		
Other analyses	17	Report other analyses done—for example, analyses of subgroups and interactions and sensitivity analyses	Section 3 and Appendix C		
Discussion					
Key results	18	Summarise key results with reference to study objectives	*Main finding of this* study in Section 4		
Limitations	19	Discuss the limitations of the study, taking into account sources of potential bias or imprecision. Discuss both direction and magnitude of any potential bias.	Section 4.1	RECORD 19.1: Discuss the implications of using data that were not created or collected to answer the specific research question(s). Include discussion of misclassification bias, unmeasured confounding, missing data and changing eligibility over time, as they pertain to the study being reported.	
Interpretation	20	Give a cautious overall interpretation of results considering objectives, limitations, multiplicity of analyses, results from similar studies and other relevant evidence.	Section 4		
Generalisability	21	Discuss the generalisability (external validity) of the study results	Section 4		
Other information					
Funding	22	Give the source of funding and the role of the funders for the present study and, if applicable, for the original study on which the present article is based.	In *Funding* section		
Accessibility of protocol, raw data and programming code				RECORD 22.1: Authors should provide information on how to access any supplemental information, such as the study protocol, raw data or programming code.	Appendix A

## Appendix C Results Without Adjustment for All‐Cause Overall Hospitalisation Rates

**TABLE C1:** Average excess (or deficit) in observed number of alcohol‐induced hospitalisations per month in the COVID‐19 pandemic period (March 2020 to April 2021) from counterfactual forecast of alcohol‐induced hospitalisations (without adjustment for all‐cause hospitalisation), overall and by sex, age and type of alcohol‐induced diagnosis.

	Estimate (95% PI)
Overall	**509 (309, 692)**
Sex	
Males	**331 (242, 422)**
Females	**163 (16, 310)**
Age	
15–34 years	**159 (112, 207)**
35–54 years	**302 (58, 531)**
55+ years	78 (−104, 242)
Diagnosis type	
CDE diseases	**222 (197, 244)**
Neuropsychiatric	**330 (91, 557)**
Poisoning	−2 (−9, 6)

*Note*: Bolded rows indicate estimates with 95% PIs that do not include 0.

Abbreviations: CDE, cardiovascular, digestive and endocrine; PI, prediction interval.
